# *BnaA01.BRC1* Negatively Regulates Branch Number and Responds to Gibberellin Signaling in *Brassica napus*

**DOI:** 10.3390/plants15121795

**Published:** 2026-06-10

**Authors:** Lujia Liu, Lanyang Ren, Xingyu Wu, Bin Zhu, Zhihui Li, Wanqing Tan, Liezhao Liu, Lili Zhang, Cunmin Qu, Ling Xie

**Affiliations:** 1 College of Agronomy and Biotechnology, Southwest University, Chongqing 400715, China; 13708319084@163.com (L.L.); rly0609@126.com (L.R.); w2530945035@163.com (X.W.); zhub51007@gmail.com (B.Z.); lizhihui@yzwlab.cn (Z.L.); sangyut@email.swu.edu.cn (W.T.); liezhao@swu.edu.cn (L.L.); drqucunmin@swu.edu.cn (C.Q.); 2 Hybrid Rapeseed Research Center of Shaanxi Province, Yangling 712100, China; zhanglili0314@nwafu.edu.cn; 3 Integrative Science Center of Germplasm Creation in Western China (Chongqing) Science City, Chongqing 401329, China

**Keywords:** *Brassica napus* L., shoot branching, *BRC1*, GA signaling, DELLA proteins

## Abstract

Plant architecture optimization is central to high-yield crop breeding. The number of branches in *Brassica napus* (*B. napus*) determines canopy structure, light use efficiency, and yield. The transcription factor *BRANCHED1* (*BRC1*) integrates multiple signals to negatively regulate branching. This study characterized five *BnaBRC1* homologs in *B. napus* via bioinformatics, expression profiling, and CRISPR/Cas9 editing. All *BnaBRC1s* contain a conserved TCP domain, and their promoters are enriched with light-responsive and hormone-responsive cis-acting elements. *BnaA01.BRC1* is highly expressed in leaves, stem nodes, roots, and siliques, and its transcription is coordinately regulated by low light, sucrose, and exogenous cytokinin, and gibberellin (GA) signals. Functional analysis showed that overexpression of *BnaA01.BRC1* suppressed branching, whereas CRISPR/Cas9-mediated knockout of *BnaBRC1* substantially increased branch number. In basal axillary buds, high *BnaBRC1* expression was accompanied by upregulation of GA-inactivating *GIBBERELLIN 2 OXIDASEs* and the GA signaling negative regulator *SPINDLY*, and no direct interaction was detected between BnaA01.BRC1 and DELLA proteins, suggesting indirect regulation of branching via GA homeostasis. Collectively, this study demonstrates the pivotal role of *BnaA01.BRC1* in branching regulation and provides a genetic resource and theoretical basis for plant architecture optimization and multi-branch germplasm innovation in *B. napus*.

## 1. Introduction

Ideal plant architecture serves as the core strategy and pivotal genetic foundation for high-yield crop breeding [1]. By optimizing plant spatial configuration and coordinating critical physiological processes including photosynthesis, nutrient partitioning, lodging resistance, high-density planting adaptability, and regulating carbon consumption from ineffective axillary branches, the crop harvest index can be effectively maximized [2,3,4,5,6,7]. *Brassica napus* (*B. napus*), the world’s second largest oilseed crop, shows close intrinsic links between branching traits and overall plant architecture. Branch number, combined with agronomic traits such as plant stature, branch angle and branch node height, determines population canopy structure, photosynthetic light utilization efficiency and final *B. napus* yield formation, relying on synergistic interaction and phenotypic trade-off effects among diverse traits [8,9]. Moreover, increasing branch number in *B. napus* improves both edible stem yield and biomass simultaneously, which is important for utilizing *B. napus* as a vegetable, forage, and green manure. Therefore, elucidating the molecular regulation of branching in *B. napus* holds substantial theoretical and practical value.

In *Arabidopsis thaliana* (*A. thaliana*), branch formation in plants is a complex developmental process. Its initiation and progression are coordinately regulated by a genetic network involving internal hormones, nutrients, and external environmental signals [10]. In tomato, brassinosteroids (BRs) are key signals that release apical dominance and antagonistically regulate lateral branching with auxin (IAA) [11]. BRs integrate multiple phytohormones, including IAA, strigolactones (SLs), gibberellin (GA), cytokinin (CK), and sugar signals. The BRs signaling transcription factor *BRASSINAZOLE RESISTANT* represses the plant specific TCP factor *BRANCHED 1*/*TEOSINTE BRANCHED 1* (*BRC1*/*TB1*), thereby relieving *BRC1* mediated inhibition of axillary bud formation [11]. In *Arabidopsis*, SLs regulate branching by inhibiting trehalose 6 phosphate signaling, an effect also dependent on *BRC1* [12]. CK promotes branching by activating *WUSCHEL2* to repress *BRC1* expression, antagonizing SLs [13,14]. Furthermore, in tomato, light-induced *LONG HYPOCOTYL 5* regulates tomato lateral branch development by suppressing *BRC1* transcription in axillary buds and activating BRs biosynthesis in lateral buds [15].

*BRC1* is the core negative regulator of branch number [16,17]. Most of the known regulatory mechanisms of *BRC1* have been elucidated in *Arabidopsis*. In *Arabidopsis*, FAR-RED ELONGATED HYPOCOTYLS 3 and FATTY ACID REDUCTASE 1 directly interact with SQUAMOSA PROMOTER BINDING PROTEIN-LIKE 9 (SPL9) and SPL15 and inhibit SPL9/15-mediated activation of *BRC1* expression, whereas three D53-like SUPPRESSOR OF MORE AXILLARY GROWTH2-LIKE proteins SMXLs (SMXL6/7/8) directly interact with SPL9/SPL15 and suppress the transactivation activity of SPL9/15 on *BRC1*, thereby promoting axillary bud growth and branching [18]. In rapeseed, *WRKY DNA-BINDING PROTEIN 28* targets and represses *BRC1*, reducing abscisic acid accumulation in leaf axils and thereby relieving bud dormancy, which leads to excessive bud outgrowth [19]. In *Arabidopsis*, BRC1 directly interacts with TCP INTERACTOR CONTAINING EAR MOTIF PROTEIN 1 to suppress its own activity, and they coregulate multiple branching-related genes such as *HOMEOBOX PROTEIN* (*HB21*), *HB40*, and *HB53* [20]. In rapeseed *brc1* mutants, expression of *Bna.HBs* and downstream *NINE-CIS-EPOXYCAROTENOID DIOXYGENASE 3s* are significantly reduced in axillary buds [9]. Furthermore, in tomato, *BRC1* reduces CK and GA accumulation by transcriptionally regulating *LONELY GUY 4*, *CYTOKININ OXIDASE 7*, *GIBBERELLIN 2 OXIDASE 4* (*GA2OX4*), and *GA2OX5*, thereby inhibiting bud growth in tomato [15]. In *Arabidopsis*, *BRC1* and *KAURENE SYNTHASE 1*, *KAURENE OXIDASE*, *KAURENOIC ACID OXIDASE*, *GIBBERELLIN 20 OXIDASE 1* (*GA20OX1*) form a positive feedback loop through the DELLA-SPL9 module, which plays a crucial role in balancing the regulation of plant height and branch number [21]. Thus, *BRC1* serves as a key hub in the complex network regulating lateral branch development.

Five *BnaBRC1* homologs in the *B. napus* genome play redundant roles in branch development [19]. *BnaBRC1* expression is significantly upregulated in middle and lower axillary buds, *BnaA01.BRC1* (*BnaA01g26700D*) shows the highest transcriptional abundance [22]. Accordingly, *BnaA01.BRC1* acts as a pivotal regulator mediating branch morphogenesis in *B. napus*. Knockout of *BRC1* via CRISPR/Cas9 gene editing can directionally create novel multi-branched *B. napus* germplasm [9]. Although *BRC1* has been extensively studied in *A. thaliana*, its functional mechanisms and regulatory networks in response to hormones, external environmental signals, and sugars in polyploid *B. napus* remain unclear. Further research on *BRC1* regulatory pathway will enrich the molecular theory of plant architecture, improve the comprehensive utilization value of *B. napus* through plant type optimization, and provide references for architecture improvement in other *Brassica* crops. To elucidate *BnaBRC1* function, this study performed bioinformatics analysis, expression profiling, and functional characterization, and preliminarily explored its regulatory role in branch development, thereby offering potential strategies and theoretical support for ideotype breeding in *B. napus*.

## 2. Results

### 2.1. Bioinformatic Characterization and Phylogenetic Analysis of BnaBRC1

**Figure 1:**
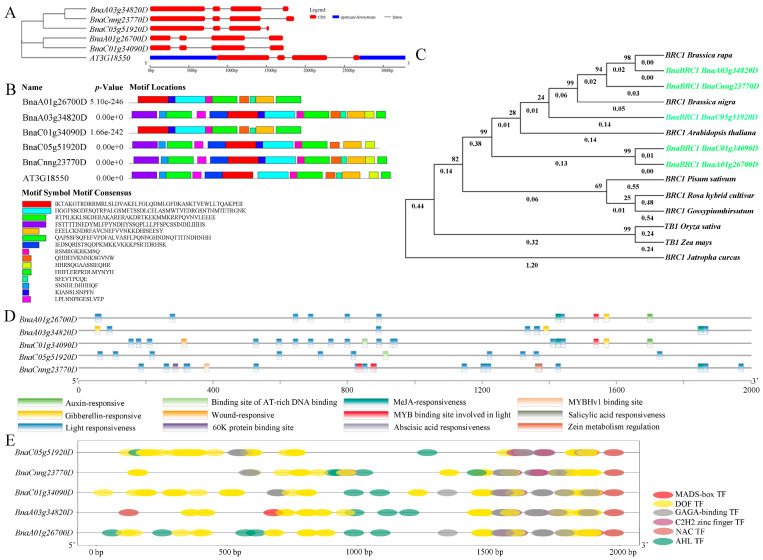
Bioinformatic characterization of BnaBRC1s in *Brassica napus*. (**A**) Gene structure of *BnaBRC1s* and *AtBRC1*. (**B**) Conserved TCP domain in BnaBRC1 proteins. (**C**) Phylogenetic relationship of BRC1 homologs across plant species. The green color indicates *BnaBRC1s*. (**D**) Predicted cis-acting elements in *BnaBRC1* promoters. (**E**) Transcription factor binding site (TFBS) prediction in *BnaBRC1* promoters.

To elucidate the structural composition, evolutionary conservation, and upstream regulatory mechanisms of the *BnaBRC1s* genes in *B. napus*, a multidimensional bioinformatics analysis was performed in this study. Gene structure analysis revealed that all *BnaBRC1s* contain four exons and three introns, exhibiting a highly conserved organization pattern with *AtBRC1* and the *BnaBRC1s* lack UTRs (Figure 1A). Protein domain analysis showed that all BnBRC1s possess a conserved TCP domain (Figure 1B). Phylogenetic analysis indicated that BnaBRC1s are closely related to BRC1 homologs from *A. thaliana*, *Brassica nigra* (*B. nigra*), and *Brassica rapa* (*B. rapa*) (Figure 1C). Promoter hormone/stress-responsive elements prediction revealed that the promoter regions of *BnaBRC1s* are enriched in light-responsive elements and various hormone-responsive elements, including IAA, GA, and jasmonates (JAs) response elements (Figure 1D). In addition, multiple cis-acting elements associated with abiotic stress responses are also distributed in these promoters (Figure 1D). Motif enrichment and prediction analyses revealed the distribution of specific transcription factor binding sites (TFBSs) in the *BnaBRC1* promoters, including binding sites for transcription factors from the MADS-box, DOF, GAGA-binding, C2H2 zinc finger, NAC, and AHL families (Figure 1E). Taken together, these results indicate that *BRC1* is relatively conserved throughout evolution and may be broadly involved in hormone signaling, plant growth and development, and abiotic stress response networks, with potential regulation by multiple transcription factors.

### 2.2. Histochemical GUS Staining and Spatiotemporal Expression Pattern of BnaA01.BRC1

GUS histochemical staining was performed to clarify the spatiotemporal expression pattern of *BnaA01.BRC1*, thereby systematically characterizing the organ-specific expression profile of this gene. The staining results revealed that *BnaA01.BRC1* was expressed at all developmental stages, and no GUS signal was detected in WT seedlings (Figure S1A). High expression levels were detected in leaves, roots, shoots, and leaf bases, with strong signals also observed at stem nodes, whereas no expression was observed in hypocotyls. Additionally, expression was also detectable in petals and siliques (Figure S1B–G).

### 2.3. Core Functional Region Identification of BnaA01.BRC1 Promoter

To further identify the core cis-acting elements of the *BnaA01.BRC1* promoter, we performed a series of 5′ deletion analysis on its promoter sequence (Figure S2A,B). GUS staining results showed that the signal weakened when the promoter was truncated to the −731 bp fragment (Figure S2B). Cis-acting element prediction revealed that the deleted region from −731 bp to −501 bp contains cis-acting elements responsive to light and multiple phytohormones, including GA, IAA, and JA (Figure S2C), which is consistent with the known feature that *BRC1* responds to various hormone signals. Notably, GUS staining became very weak after truncation to −501 bp. Therefore, we propose that the region from −731 bp to −501 bp is the essential core domain required for maintaining the basal activity of the *BnaA01.BRC1* promoter.

### 2.4. Cis-Responsive Patterns of BnaA01.BRC1 Under Diverse Exogenous Signal Treatments

**Figure 2:**
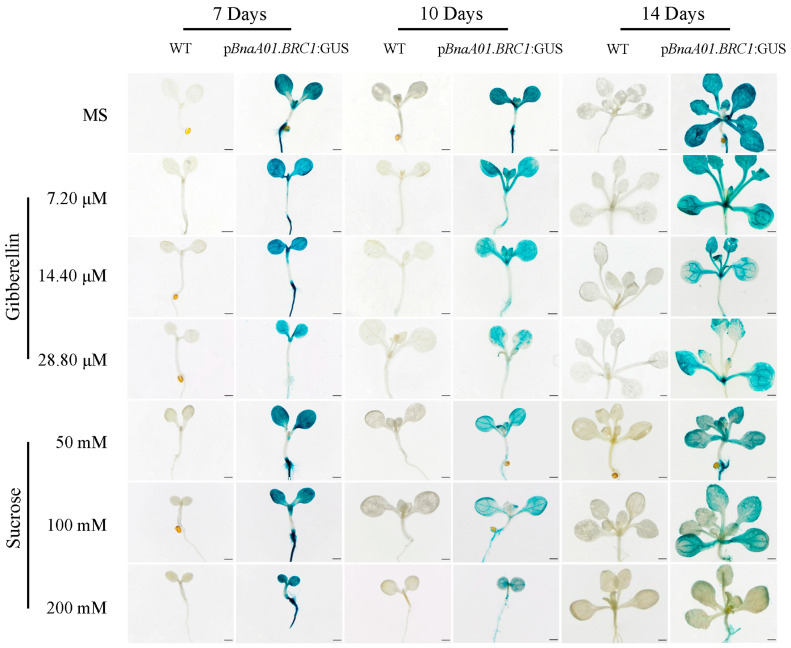
Exogenous GA_3_ and sucrose suppress *BnaA01.BRC1* promoter activity (Bar = 1000 μm).

To systematically characterize the response patterns of the *BnaA01.BRC1* promoter to various exogenous environmental and hormonal signals, based on the predicted cis-acting elements within its key regions, p*BnaA01.BRC1*::GUS transgenic plants were subjected to low-light treatment as well as graded concentrations of IAA, 6-benzylaminopurine (6-BA), GA, and sucrose, respectively. Low-light stress markedly induced hypocotyl elongation in seedlings, and this phenotype progressively intensified with prolonged treatment. Compared with normal light conditions, low-light treatment significantly expanded the GUS staining range and enhanced the staining intensity (Figures S3 and S5). Accordingly, it is proposed that *BnaA01.BRC1* responds to low-light signals by enhancing its own promoter transcriptional activity, thereby upregulating *BnaA01.BRC1* expression. Unlike low light, IAA treatment showed no obvious enhancement of GUS staining intensity under the tested concentrations, and no dose-dependent effect was observed (Figure S4). In contrast, 6-BA, GA, and sucrose all suppressed GUS signals in a concentration-dependent manner, with reduced staining area and weakened intensity as the concentration increased (Figure 2 and Figures S4 and S5). Collectively, these results demonstrate that *BnaA01.BRC1* broadly responds to light, multiple phytohormones, and sugar signals, thereby coordinately regulating gene expression and shoot branching development.

### 2.5. Subcellular Localization Analysis of BnaA01.BRC1

Secondary structure analysis of BnaA01.BRC1 revealed that the protein predominantly adopts an α-helical conformation (Figure S6A). The predicted tertiary structure model further indicated that the three-dimensional folding of BnaA01.BRC1 is conserved and stable, with the spatial conformation of its core TCP functional domain being highly conserved (Figure S6B). Subcellular localization assays using *A. thaliana* protoplasts showed that the green fluorescence signal of the BnaA01.BRC1-GFP fusion protein completely overlapped with that of the nuclear marker, confirming that BnaA01.BRC1 is specifically localized to the nucleus, which is consistent with the subcellular distribution feature of a transcription factor (Figure S6C).

### 2.6. Functional Genetic Analysis of BnaA01.BRC1 Mediating Plant Branch Development

**Figure 3:**
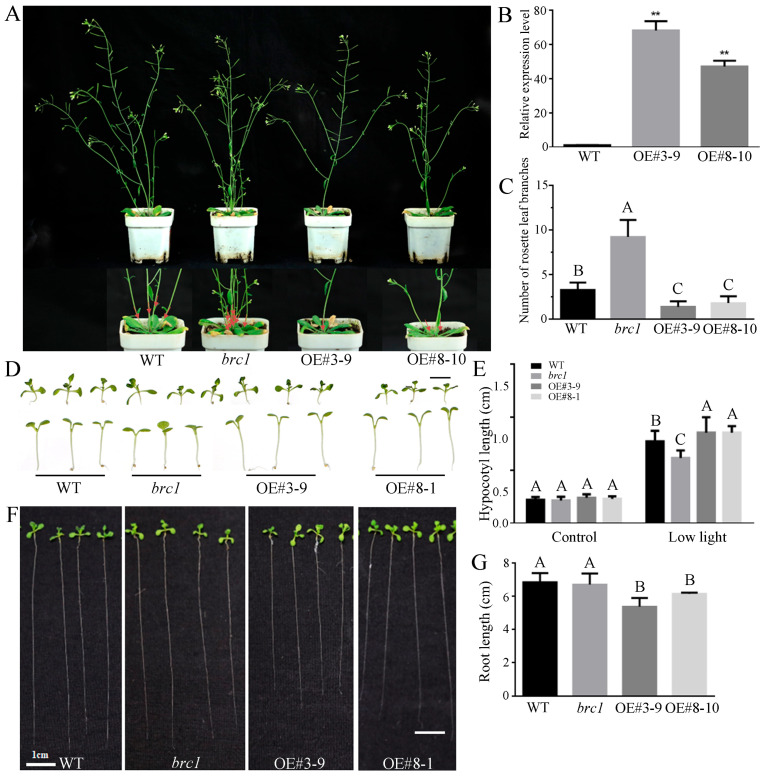
Heterologous overexpression of *BnaA01.BRC1* in *Arabidopsis thaliana* affects branching, hypocotyl elongation, and root growth. (**A**) Rosette branch phenotype. (**B**) Relative expression of *BnaA01.BRC1* in overexpression lines (** *p* ≤ 0.01). (**C**) Statistical analysis of rosette branch number. (**D**) Hypocotyl phenotype under low light (Bar = 2000 μm). (**E**) Hypocotyl length quantification. (**F**) Root growth phenotype (Bar = 1 cm). (**G**) Root length quantification. All data are presented as mean ± SE (*n* ≥ 20 plants per genotype). Different capital letters indicate significant differences among groups (*p* < 0.05).

*BnaA01.BRC1* was heterologously expressed in *A. thaliana* to determine its role in branch formation. At full-bloom stage, the rosette branch numbers were counted in WT, *brc1*, and *BnaA01.BRC1* heterologous overexpression lines (OE#3-9 and OE#8-10). The results showed that, compared with WT, overexpression lines exhibited significantly fewer rosette branches, whereas the *brc1* displayed significantly more rosette branches (Figure 3A). RT-qPCR analysis revealed that the expression levels of *BnaA01.BRC1* in the OE#3-9 and OE#8-10 lines were 68-fold and 47-fold higher than that in WT, respectively (Figure 3B). The rosette branch number statistics further indicated that *brc1* had significantly more branches than WT, while both OE#3-9 and OE#8-10 had significantly fewer branches than WT (Figure 3C). These phenotypic observations were highly consistent with the reported function of *AtBRC1* in negatively regulating lateral branching in *A. thaliana*, confirming that *BnaA01.BRC1* shares a conserved branch-suppressing function with its *A. thaliana* homolog. Since *BnaA01.BRC1* senses light signal changes (Figures S3 and S5), heterologous *BnaA01.BRC1* overexpression lines and *brc1* were subjected to low-light treatment. The results showed that under low-light conditions, the overexpression lines exhibited markedly elongated hypocotyls, whereas *brc1* showed the opposite trend (Figure 3D,E). These lines of evidence suggest that elevated *BnaA01.BRC1* expression promotes stem organ elongation under low light, thereby affecting overall plant growth. Root length measurements revealed that *BnaA01.BRC1* heterologous overexpression lines had significantly shorter roots than both WT and *brc1* (Figure 3F,G), indicating that *BnaA01.BRC1* overexpression may inhibit root development.

**Figure 4:**
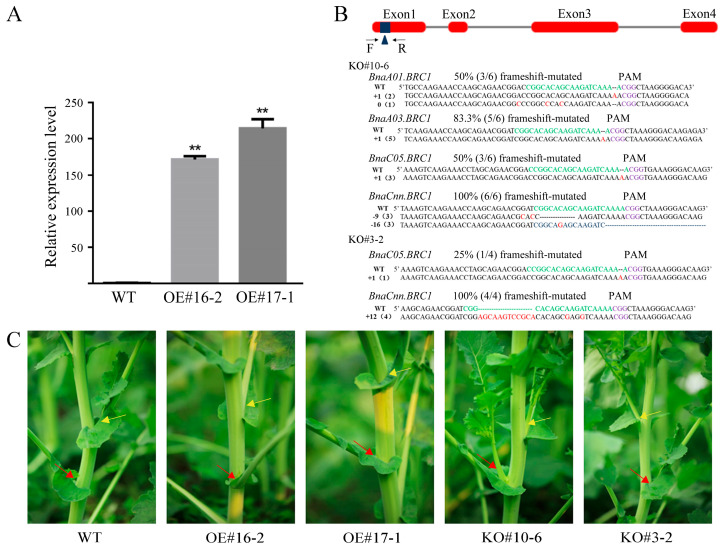
Overexpression and CRISPR/Cas9 knockout of *BnaBRC1* alter branch number in *Brassica napus*. (**A**) Relative expression of *BnaA01.BRC1* in overexpression lines (** *p* ≤ 0.01). (**B**) Editing results of *BnaBRC1* knockout lines. Green font indicates the CRISPR/Cas9 knockout target site, purple font indicates the protospacer adjacent motif (PAM) structure, and red font indicates the edited sequence. (**C**) Development of middle and lower axillary buds in *BnaA01.BRC1* overexpression lines and *BnaBRC1* knockout lines at initial flowering stage. Yellow arrows indicate middle axillary buds, and red arrows indicate lower axillary buds.

Transgenic lines overexpressing *BnaA01.BRC1* and CRISPR/Cas9-based knockout plants were generated for phenotypic observation (Figure 4A,B). Phenotypic analysis at the initial flowering stage revealed that axillary buds at the middle and lower positions developed normally into branches in WT. In the overexpression lines, most axillary buds at the middle and lower positions failed to develop into branches, whereas in the knockout plants, axillary buds at the corresponding positions developed normally into branches (Figure 4C). These results indicate that high expression of *BnaBRC1* is a critical factor inhibiting axillary bud outgrowth into branches at the middle and lower positions in *B. napus*.

### 2.7. Molecular Mechanism of BnaA01.BRC1 Involved in the GA Signaling Pathway

**Figure 5:**
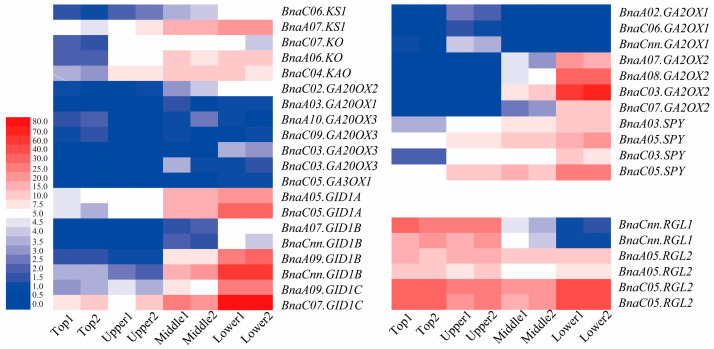
Expression patterns of GA signaling-related genes in axillary buds of *Brassica napus*.

The expression level of *BnaBRC1* in *B. napus* axillary buds increases as bud position descends, with the highest expression observed in the lowest axillary buds, reaching up to a hundred-fold difference [22]. Using previously obtained transcriptome data from axillary buds at different positions in *B. napus*, genes involved in the GA signaling pathway were analyzed [22]. The results showed that multiple genes participating in GA biosynthesis, including *GA20OX* and *GIBBERELLIN 3 OXIDASE* (*GA3OX*), as well as several homologs of *GIBBERELLIN INSENSITIVE DWARF1* (*GID*) involved in GA signal transduction, exhibited the same expression trend: lower bud positions were associated with higher expression levels (Figure 5). Interestingly, *GA2OX1* and *GA2OX2*, which are involved in GA inactivation, and *SPINDLY* (*SPY*), a negative regulator of GA signaling, also displayed a similar expression pattern (Figure 5). In contrast, genes encoding DELLA proteins, the core repressors of the GA signaling pathway, such as *RGA-LIKE 1* (*RGL1*) and *RGL2*, exhibited the opposite expression pattern (Figure 5).

**Figure 6:**
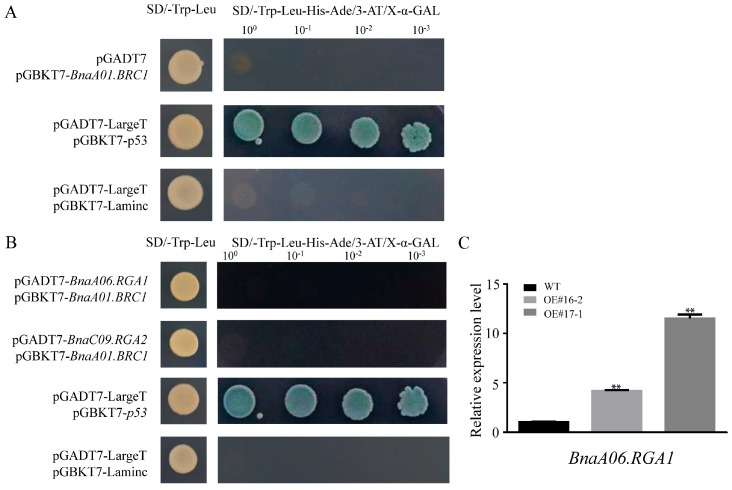
BnaA01.BRC1 does not directly interact with DELLA proteins but upregulates *BnaA06.RGA1* expression. (**A**) Transcriptional activation activity assay. (**B**) Yeast two-hybrid assays showing no interaction with BnaA06.RGA1 or BnaC09.RGA2. (**C**) Relative expression of *BnaA06.RGA1* in overexpression lines. (** *p* ≤ 0.01).

Transcriptional activation activity assays revealed that BnaA01.BRC1 had no autoactivation activity (Figure 6A). Given that the core region of the *BnaA01.BRC1* promoter contains GA responsive cis-acting elements and that this gene is transcriptionally responsive to exogenous GA, yeast two-hybridization assays were further performed to test the interactions of the BnaA01.BRC1 protein with the DELLA family proteins REPRESSOR OF GA (BnaA06.RGA1) and RESTORATION ON GROWTH ON AMMONIA 2 (BnaC09.RGA2), respectively. The pairwise yeast two-hybrid assays (Y2H) confirmed that BnaA01.BRC1 does not directly interact with BnaA06.RGA1 or BnaC09.RGA2 (Figure 6B). In *BnaA01.BRC1* overexpression lines, *BnaA06.RGA1* transcript levels were significantly elevated (Figure 6C). These results indicate that *BnaA01.BRC1* acts indirectly in the GA signaling pathway, likely through modulation of GA homeostasis or downstream transcriptional cascades, leading to spatiotemporal regulation of DELLA family gene expression and consequently affecting axillary bud branching in *B. napus*.

## 3. Discussion

In past decades, the regulatory mechanisms of plant architecture have been systematically elucidated [23,24,25,26]. Branch number, a key determinant of plant morphology and yield, has received increasing attention [27,28]. The development of axillary buds into branches involves multiple layers of regulation [29,30]. Studies in multiple species have shown that *BRC1* precisely controls branch number by inhibiting axillary bud outgrowth [9,31,32,33]. In this study, transgenic and CRISPR/Cas9 technologies were used to generate *BnaA01.BRC1* overexpression and knockout lines. Phenotypic analysis revealed that *BnaA01.BRC1* overexpression suppressed branch growth, whereas knockout significantly increased branch number (Figure 3 and Figure 4). These results indicate that *BnBRC1* negatively regulates branching in rapeseed, consistent with the functions of *AtBRC1* and *OsTB1* in suppressing branching/tillering, suggesting a highly conserved role among them [34,35]. Therefore, targeted manipulation of *BRC1* via gene editing can generate multi-branch rapeseed germplasm, optimize plant architecture, and enhance its multiple utilities for vegetable, forage, and green manure purposes.

*BRC1* is a central integrator of hormonal, nutritional, and environmental signals regulating lateral branch development across multiple species [36,37]. *BnaA01.BRC1* in rapeseed responds to IAA, CK, GA, sucrose, and light signals (Figure 2 and Figures S3–S5). Light not only drives photosynthesis to supply sucrose but also acts as a signal for photomorphogenesis [38]. In the phytochrome A signaling pathway, FHY3/FAR1 interacts with SPL9/15 and suppress their transcriptional activation of *BRC1*, thereby promoting branching [18]. In this study, *BnaA01.BRC1* responds to low light by enhancing its own promoter transcriptional activity (Figures S3 and S5). Low light inhibits branching and upregulates *BRC1* expression [39,40]. During the shade avoidance response, plants elevate leaves, elongate stems, and suppress branching to capture more light, accompanied by increased *BRC1* expression [34]. Under low light, *BnaA01.BRC1* overexpressing *A. thaliana* seedlings exhibited longer hypocotyls than the WT, whereas *brc1* mutants showed shorter hypocotyls (Figure 3). Elevated *TB1* expression is known to inhibit internode elongation [41]. These results indicate that *BnaA01.BRC1* responds to changes in light intensity, promotes stem elongation, and inhibits axillary bud outgrowth, thereby participating in light-dependent regulation of axillary branching and plant architecture. Furthermore, *BRC1* serves as a key node integrating sugar and hormonal signals to regulate branching [12]. Exogenous sucrose treatment suppressed *BnaA01.BRC1* expression, suggesting that *BnaA01.BRC1* may regulate branching in rapeseed by responding to sugar signals.

IAA regulates *BRC1* expression in axillary buds by antagonizing CK and SL [11,42,43]. High CK levels promote bud activation by downregulating *BRC1* [12]. In this study, CK suppressed *BnaA01.BRC1* promoter activity in a dose-dependent manner, whereas IAA treatment did not cause a significant change under the tested concentrations, possibly due to insufficient IAA concentration (Figures S4 and S5). It is speculated that CK regulates bud activity and promote branching in rapeseed via *BRC1* downregulation. In addition to CK, GA also participates in *BRC1* regulation [15,44,45]. Exogenous GA treatment suppressed *BnaA01.BRC1* promoter-driven GUS signals, indicating GA-mediated repression of this gene (Figure 2 and Figure S5). Low GA content is required for axillary bud formation in *A. thaliana*, and ectopic *GA20OX2* expression in leaf axils elevates GA levels and inhibits axillary meristem initiation [46]. GA2OX1 and GA2OX2 are key enzymes that inactivate GA, whereas *SPY* negatively regulates GA responses [47,48]. In basal axillary buds of rapeseed with high *BnaBRC1* expression, *GA2OX1*, *GA2OX2* and *SPY* were significantly upregulated (Figure 5). This may lead to reduced local GA activity and attenuated downstream signaling in basal buds. *GA20OX* and *GA3OX* are key genes involved in GA synthesis, whereas *GID* is a key gene involved in GA signaling [49,50]. *GA20OX*, *GA3OX*, and *GID* were also upregulated, suggesting a higher potential for GA signaling output in basal buds (Figure 5). Collectively, the transcriptional regulation of GA signaling exhibits strong spatial specificity in rapeseed axillary buds. The coordinated upregulation of both promoting and repressing modules indicates that basal axillary buds are not in a simple on or off state but rather in a highly sensitive and finely tuned standby or homeostatic state. It is proposed that basal buds establish a robust GA signaling system while simultaneously deploying *GA2OX* and *SPY* as “brakes” to prevent premature or excessive bud outgrowth, thereby maintaining a dormant state with low activity and high potential.

DELLA proteins are core repressors of GA signaling [51]. *RGL1* and *RGL2*, which encode DELLA proteins, are partially redundant but distinct negative regulators of GA responses [52,53]. *RGL2* and *RGL1* exhibited a decreasing gradient from upper to lower axillary buds in rapeseed. This suggests that low DELLA levels may be a key molecular event for releasing basal bud dormancy, but whether a bud eventually sprouts depends on the net balance of promoting and repressing factors within the GA signaling network and their integration with other hormonal signals. Yeast two-hybrid assays showed no direct interaction of BnaA01.BRC1 with DELLA proteins BnaA06.RGA1 and BnaC09.RGA2, and *BnaA06.RGA1* was upregulated in *BnaA01.BRC1* overexpressing plants (Figure 6). *BRC1* has been shown to reduce GA accumulation by upregulating *GA2OX4* and *GA2OX5*, thereby inhibiting bud outgrowth [15,36]. It is therefore speculated that *BnaA01.BRC1* in rapeseed reduces GA content and inhibits bud development by upregulating *GA2OXs*, while also directly or indirectly increasing *BnaA06.RGA1* expression to promote DELLA accumulation and further lower GA levels. DELLA proteins interact with SPL9 and modulate its DNA-binding affinity, forming a DELLA-SPL9-LAS-GA2OX4 feedback module that establishes a low GA microenvironment in leaf axils to precisely control axillary bud formation in *A. thaliana* [46]. Whether the upregulated *BnaA06.RGA1* in *BnaA01.BRC1* overexpressing plants participates in axillary bud formation via this module or through crosstalk with other hormones requires further investigation.

## 4. Materials and Methods

### 4.1. Plant Materials and Growth Conditions

The transgenic lines of *A. thaliana* (Col-0) and *B. napus* used in this study were in the Col-0 and J9709 genetic backgrounds, respectively. The *A. thaliana brc1* (SALK_091920) was obtained from AraShare (Airuosha Biotech, Fuzhou, China). *N. benthamiana* was used for subcellular localization of BnaA01.BRC1. Transgenic *B. napus* plants were grown in the transgenic plant greenhouse of Southwest University, with field management following standard agricultural practices. Transgenic *B. napus* materials for successive generation advancement and phenotypic analysis were cultivated in an artificial climate chamber under the following conditions: a light intensity of 15,000 lx, relative humidity of 70–80%, and a photoperiod of 14 h light (24 °C)/10 h dark (22 °C) per cycle. *N. benthamiana* and *A. thaliana* were also grown in an artificial climate chamber under the following conditions: a light intensity of 15,000 lx, relative humidity of 75%, and a photoperiod of 16 h light (22 °C)/8 h dark (20 °C).

### 4.2. Bioinformatic Characterization of BnaBRC1

The full-length gene sequences, 2000-bp promoter sequences, and protein sequences of all *BnaBRC1* homologous genes were downloaded from the BnPIR: *Brassica napus* pan-genome information resource (http://cbi.hzau.edu.cn/bnapus/) (accessed on 28 November 2025), and the full-length gene sequence and protein sequence of *AtBRC1* gene were downloaded from TAIR (https://www.arabidopsis.org/) (accessed on 28 November 2025). The online tool GSDS 2.0 (http://gsds.gao-lab.org/) (accessed on 2 December 2025) was used to visualize the gene structure. Motif prediction analysis of BnaBRC1 and AtBRC1 was performed using NCBI Conserved Domains (https://www.ncbi.nlm.nih.gov/Structure/cdd/wrpsb.cgi) (accessed on 5 December 2025), and the sequence motifs were visualized using MEME (https://meme-suite.org/meme/) (accessed on 6 December 2025). Promoter cis-acting element prediction was performed using PlantCare (http://bioinformatics.psb.ugent.be/webtools/plantcare/html/) (accessed on 28 November 2025), and the identified hormone- and stress-responsive elements were visualized using TBtools (https://github.com/CJ-Chen/TBtools/releases) (accessed on 28 November 2025). For modern TFBS motif analysis, the MEME Suite tools were employed. Specifically, Analysis of Motif Enrichment (https://meme-suite.org/meme/tools/ame) (accessed on 28 May 2026)was used for motif enrichment analysis against the JASPAR CORE (2026) non-redundant plant motif database, using the total odds score method and the ranksum test. TFBSs were predicted using Find Individual Motif Occurences (https://meme-suite.org/meme/tools/fimo) (accessed on 28 May 2026). The distribution of predicted TFBSs (q-value < 0.05) was visualized using ggplot2 in R. Protein sequences of BRC1/TB1 from *B. rapa*, *B. nigra*, *A. thaliana*, *Pisum sativum*, *Rosa hybrid cultivar*, *Gossypium hirsutum*, *Oryza sativa*, *Zea mays*, and *Jatropha curcas* were batch-downloaded from the National Center for Biotechnology Information (https://www.ncbi.nlm.nih.gov/) (accessed on 11 December 2025). Sequence alignment was performed using MEGA7.0, and a phylogenetic tree was subsequently constructed. Transmembrane domain prediction of the BnaA01.BRC1 protein sequence was conducted using TMHMM-2.0 (https://services.healthtech.dtu.dk/services/TMHMM-2.0/) (accessed on 12 December 2025). Tertiary structure prediction of the protein was performed using the SWISS-MODEL server (https://swissmodel.expasy.org/) (accessed on 13 December 2025), and the optimal model was selected based on the prediction results.

### 4.3. Plasmid Construction, A. thaliana and B. napus Transformation

To generate *BnaA01.BRC1* overexpression transgenic plants, the full-length CDS of *BnaA01.BRC1* (excluding the stop codon) was amplified and cloned into the expression vector via the *Bam*HI and *Hind*III sites, yielding the p35S::*BnaA01.BRC1*-DsRed construct. For CRISPR/Cas9-based knockout, a 19 bp gRNA (5′-CCGGCACAGCAAGATCAAAA-3′) targeting the five homologous *BnaBRC1* genes was designed and inserted into the p2×35S-dpCas9-atU6-Grna-35S-Hy vector. Subsequently, the resulting recombinant plasmids were introduced into *Agrobacterium tumefaciens* and transformed into *A. thaliana* and *B. napus* (cultivar J9709) via the *Agrobacterium*-mediated method. The transgenic plants were verified by PCR, and the expression level of *BnaA01.BRC1* in the overexpression lines was further confirmed by RT-qPCR. For the knockout lines, genomic DNA was extracted and the targeted region was amplified with primers flanking the gRNA cleavage site; the PCR products were then sequenced to confirm successful editing. All primers used for constructing the fusion expression vectors are listed in Supplementary Table S1.

### 4.4. Histochemical GUS Staining

To generate the p*BnaA01.BRC1*::GUS reporter construct, the 1500 bp promoter fragment of *BnaA01.BRC1* was amplified from genomic DNA of the cultivar Zhongshuang 11 (ZS11) and inserted into the binary vector pCAMBIA1305.1 via *Hind*III and *Nco*I restriction sites (Supplementary Table S1). A series of 5′ truncated promoter fragments (1411, 1231, 1001, 731, 501, 371, 111, and 1411 306 bp) were generated in parallel to determine the core promoter region and introduced into the same vector to produce the corresponding p*BnaA01.BRC1*::GUS fusion constructs (Supplementary Table S1). All resultant plasmids were introduced into *Agrobacterium tumefaciens* strain GV3101. The recombinant p*BnaA01.BRC1*::GUS (full-length promoter) was then transformed into *A. thaliana* by the floral dip method. For the obtained positive transgenic lines, T3 progeny plants were used for histochemical GUS staining according to the instructions of the GUS Staining Kit (Coolaber, Beijing, China). Samples were incubated at 37 °C in the dark for 16 h. Stained tissues were photographed using a Nikon SMZ1500 stereomicroscope (Nikon, Tokyo, Japan). The truncated promoter constructs were separately delivered into *N. benthamiana* leaves via *Agrobacterium* mediated infiltration, followed by GUS analysis under the same staining protocol.

### 4.5. Different Exogenous Hormones, Sucrose, and Low-Light Treatments

Treatment media were prepared using MS medium supplemented with IAA (1.14, 2.85, 5.70 μM), 6-BA (4.44, 8.88, 22.2 μM), GA3 (7.20, 14.4, 28.8 μM), and sucrose (50, 100, 200 mM) under normal light conditions. Meanwhile, MS medium was placed under low-light conditions (light intensity < 80 lx). The p*BnaA01.BRC1*::GUS seedlings were grown under each of the above conditions, and at 7, 10, and 14 days of age, GUS staining was performed on the seedlings using the same method, followed by observation under a stereomicroscope with consistent illumination and exposure settings. Each treatment was repeated three times, with no less than 5 plants in each group. For quantitative analysis of GUS staining intensity, the acquired images were processed using ImageJ (v1.54p, National Institutes of Health, Bethesda, MA, USA). Briefly, each image was split into its RGB channels, and only the blue channel was retained to specifically represent the GUS signal. The Mean Gray Value calculated as RawIntDen/Area was measured for the entire region of each seedling. For each treatment group, data from at least 5 biological replicates were collected, and the experiment was repeated three times. Statistical significance between each treatment and the control was evaluated using Student’s *t*-test.

### 4.6. Subcellular Localization Analysis

To create a C-terminal fusion of BnaA01.BRC1 with GFP, the full-length cDNA of *BnaA01.BRC1* was amplified from ZS11 (Supplementary Table S1). The resulting fragment was cloned into the *Spe*I and *Bam*HI enzyme sites of pAN580 vector. The plasmids pAN580 (empty control) and pAN580-BnaA01.BRC1-GFP were transformed into GV3101. The recombinant plasmid was transiently expressed in *A. thaliana* protoplasts using polyethylene glycol-mediated transformation, and BnaA01.BRC1 subcellular localization analysis was performed by laser confocal microscopy.

### 4.7. Phenotypic Analysis

Following the aforementioned experimental procedures, WT, *brc1* and heterologous *BnaA01.BRC1*-overexpressing transgenic *A. thaliana* lines were treated under low-light conditions for 14 days, and their hypocotyl lengths were subsequently subjected to statistical analysis. The identical plant seedlings were cultivated on MS medium under normal growing conditions for 12 days, and their root lengths were subsequently subjected to statistical analysis. At the full flowering stage, the branch numbers of the same plants were observed and counted. At least 20 plants per genotype were used for statistical analysis. All data are presented as mean ± standard error (SE). One-way analysis of variance (ANOVA) followed by Tukey’s multiple comparisons test was performed to evaluate the statistical significance of differences among genotypes. Different capital letters in the figures indicate significant differences at *p* < 0.05. At the initial flowering stage, branch development was observed in transgenic *B. napus* plants overexpressing *BnaA01.BRC1* and *BnaBRC1* knockout lines, using J9709 as a control.

### 4.8. Yeast Two-Hybrid

For Y2H assays, the coding sequence of *BnaA01.BRC1* was amplified and ligated into the bait vector pGBKT7 using the *Nco*I and *Bam*HI sites. The prey vectors pGADT7 carrying the full-length CDSs of *BnaA06.RGA1* or *BnaC09.RGA2* were constructed via *Eco*RI and *Bam*HI cloning, respectively. All primers used for vector construction are listed in Supplementary Table S1. The resulting bait and prey plasmids were co-transformed into yeast strain Y2HGold using the Yeastmaker™ Yeast Transformation System 2 (Clontech, Mountain View, CA, USA) following the manufacturer’s protocol. Transformants were plated on SD/-Trp/-Leu for selection, and pairwise interactions were assessed on SD/-Trp/-Leu/-His/-Ade medium.

### 4.9. RNA Extraction, cDNA Synthesis and Quantitative RT-PCR

Total RNA was isolated from 4-week-old seedlings with the EZ-10 DNAaway RNA Mini-Prep Kit (Sangon Biotech, Shanghai, China) following the manufacturer’s protocol. First-strand cDNA was reverse-transcribed from the RNA using the PrimeScript™ RT reagent kit with gDNA Eraser (TaKaRa, Kusatsu, Japan), and quantitative real-time PCR (RT-qPCR) was carried out with TB Green^®^ Premix Ex Taq™ II (Takara) on a CFX96 Real-Time PCR Detection System. All primers are listed in Supplementary Table S1. Each sample had three independent biological replicates, and PCR amplifications were run according to the kit manufacturer’s instructions. *Bna.Actin7* and *AtActin2* served as internal controls for *B. napus* and *A. thaliana*, respectively. Relative transcript levels were calculated using the 2^−ΔΔCT^ method [54].

### 4.10. Transcriptome Analysis

Transcriptome data of *B. napus* axillary buds were retrieved from our published study [22]. Briefly, sample collection, total RNA isolation, cDNA library preparation, RNA-seq and bioinformatics analysis were performed as described previously. The original raw transcriptome data are available at the Sequence Read Archive of the National Center for Biotechnology Information (PRJNA523473).

## Data Availability

The data presented in this study are available in the article and Supplementary Materials. The promoter sequences and protein sequences of BnaBRC1 homologs can be accessed from the BnPIR database (http://cbi.hzau.edu.cn/bnapus/).

## Supplementary Materials

The following supporting information can be downloaded at: https://www.mdpi.com/article/10.3390/plants15121795/s1, Figure S1. Spatiotemporal expression pattern of *BnaA01.BRC1* revealed by GUS staining. (A) GUS staining in wild-type (negative control) and p*BnaA01.BRC1*:GUS transgenic *Arabidopsis* seedlings at different developmental stages (7, 10, 14 and 21 days after germination, from left to right); (B–G) Tissue-specific expression of *BnaA01.BRC1* in rosette leaves, axillary buds, axillary branches, apical buds, siliques and roots of 35-day-old p*BnaA01.BRC1*:GUS *Arabidopsis* plants. Bar = 1000 μm. Figure S2. Core functional region identification of *BnaA01.BRC1* promoter by 5′ deletion analysis. (A) Electrophoretic detection of serially truncated *BnaA01.BRC1* promoter fragments; (B) GUS staining of each truncation; (C) Predicted cis-acting elements in the −731 to −1 bp region. Figure S3. Low light treatment induces *BnaA01.BRC1* promoter activity and promotes hypocotyl elongation (Bar = 1000 μm). Figure S4. Exogenous IAA and 6-BA regulate *BnaA01.BRC1* promoter activity in a dose-dependent manner (Bar = 1000 μm). Figure S5. Quantitative analysis of GUS staining intensity in p*BnaA01.BRC1*:GUS transgenic seedlings (**, *p* < 0.01; *, *p* < 0.05; ns, not significant). Figure S6. Protein structure and subcellular localization of BnaA01.BRC1. (A) Predicted secondary structure; (B) Predicted tertiary structure with conserved TCP domain. The core TCP functional domain is highlighted in red in the model; (C) Subcellular localization showing nuclear enrichment in *Arabidopsis* protoplasts (Bar = 10 μm). Table S1. Primers used in this study.
