# Supplementary material for: BnaA01.BRC1 Negatively Regulates Branch Number and Responds to Gibberellin Signaling in Brassica napus
Source: Plants (Basel). 2026 Jun 10;15(12):1795. doi: 10.3390/plants15121795 (PMC13306499; doi:10.3390/plants15121795)
Supplement: Supplementary file 1 [file plants-15-01795-s001.zip › Supplementary Figures.pdf]

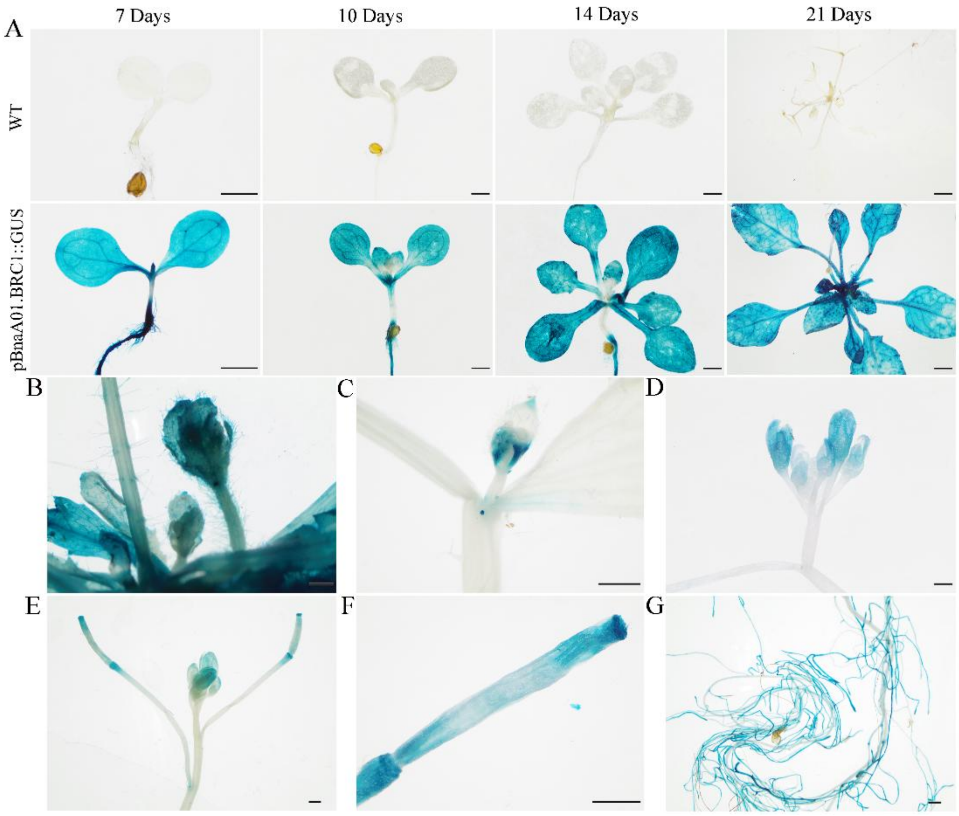

Figure S1. Spatiotemporal expression pattern of *BnaA01.BRC1* revealed by GUS staining. (A) GUS staining in wild-type (negative control) and p*BnaA01.BRC1*:GUS transgenic *Arabidopsis* seedlings at different developmental stages (7, 10, 14 and 21 days after germination, from left to right). (B–G) Tissue-specific expression of *BnaA01.BRC1* in rosette leaves, axillary buds, axillary branches, apical buds, siliques and roots of 35-day-old p*BnaA01.BRC1*:GUS *Arabidopsis* plants. Bar = 1000 μm.

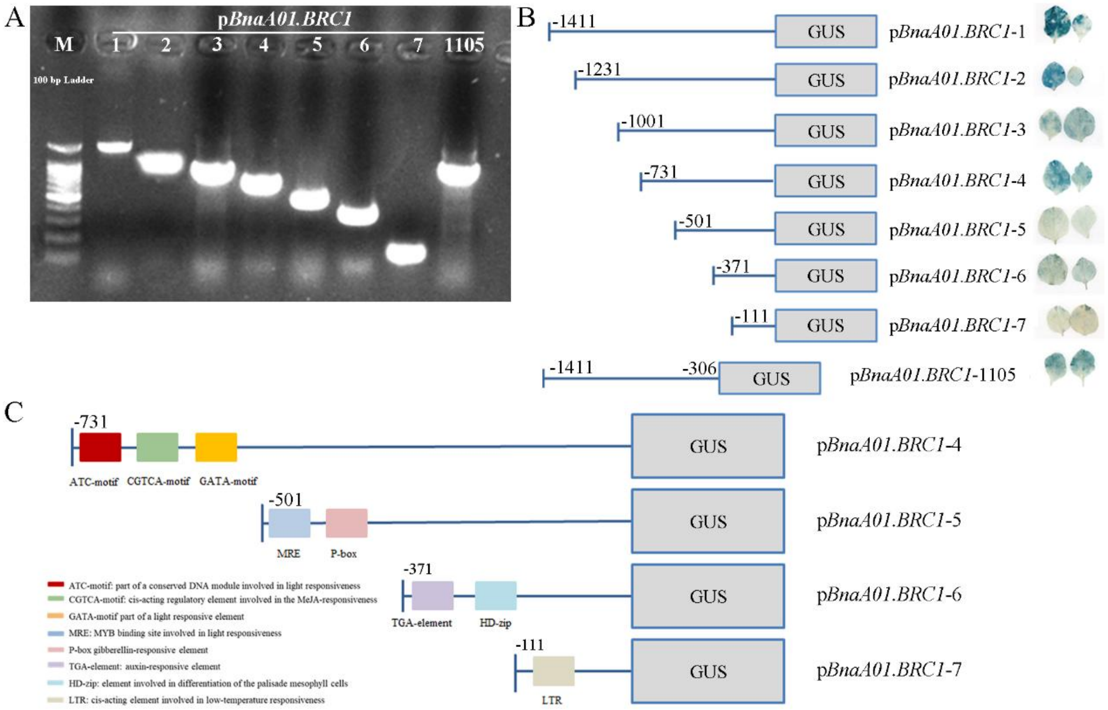

Figure S2. Core functional region identification of *BnaA01.BRC1* promoter by 5′ deletion analysis. (A) Electrophoretic detection of serially truncated *BnaA01.BRC1* promoter fragments. (B) GUS staining of each truncation. (C) Predicted cis-acting elements in the -731 to -1 bp region.

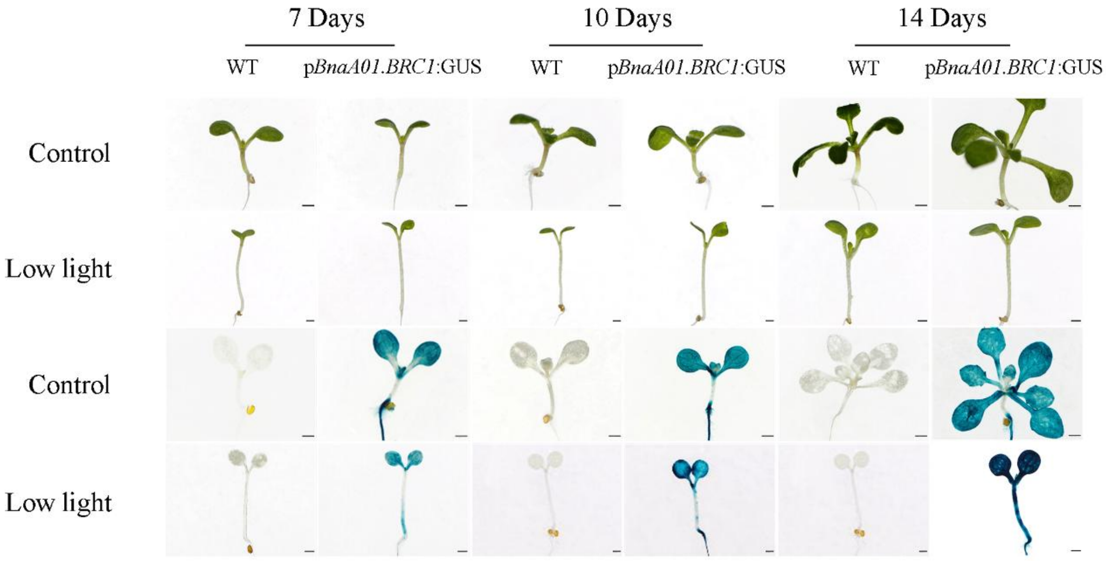

Figure S3. Low light treatment induces *BnaA01.BRC1* promoter activity and promotes hypocotyl elongation (Bar = 1000 μm).

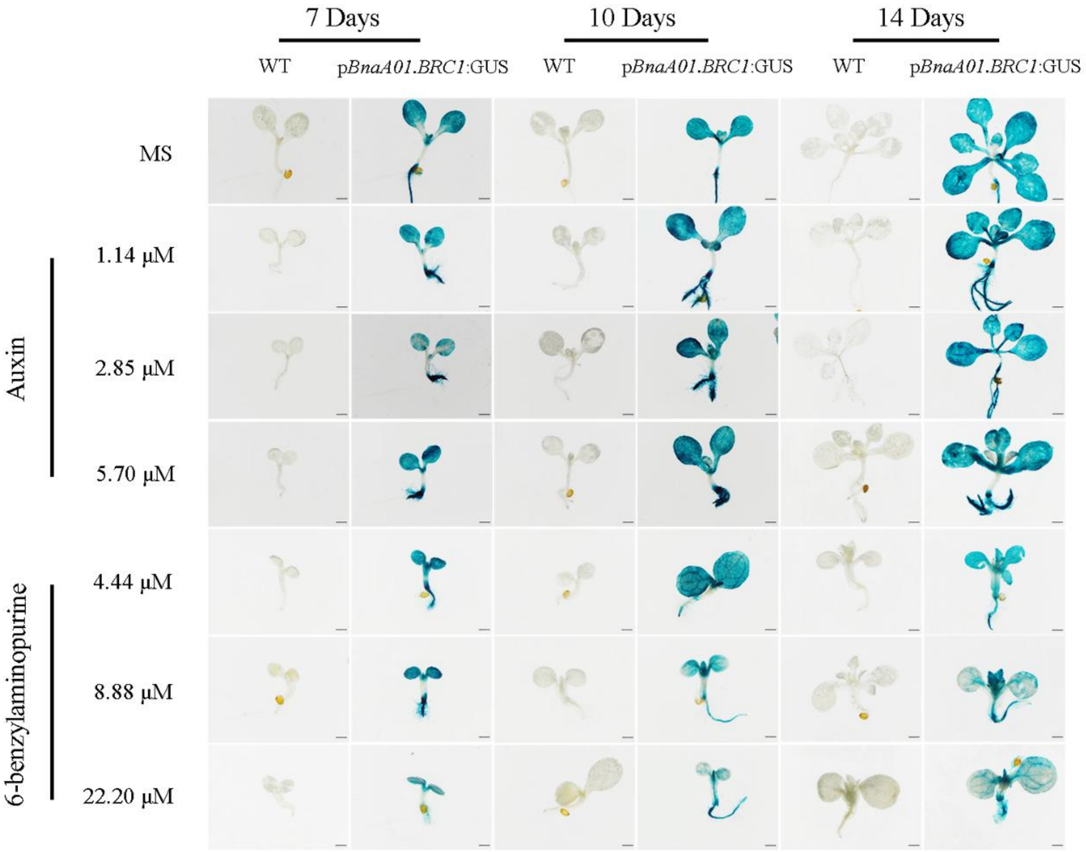

Figure S4. Exogenous IAA and 6-BA regulate *BnaA01.BRC1* promoter activity in a dose-dependent manner (Bar = 1000 μm).

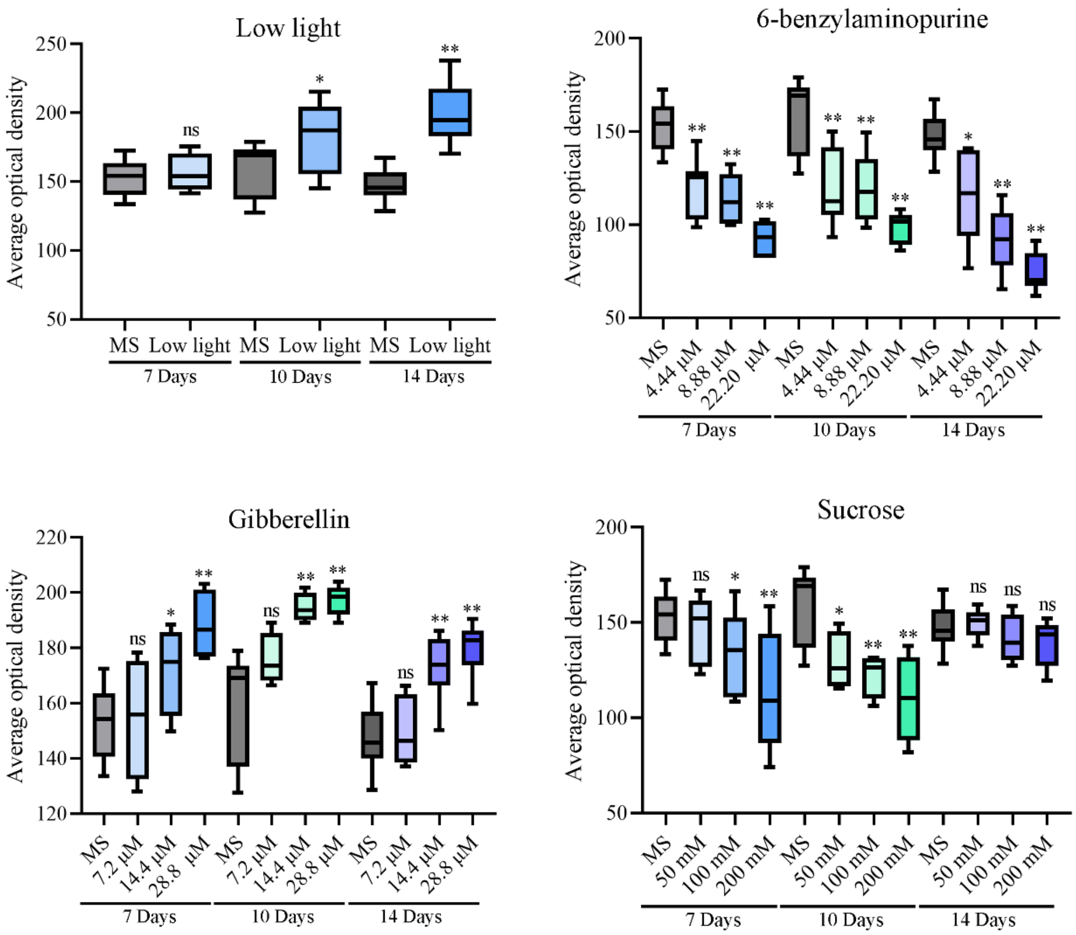

Figure S5. Quantitative analysis of GUS staining intensity in p*BnaA01.BRC1*:GUS transgenic seedlings (**, $p < 0.01$; *, $p < 0.05$; ns, not significant).

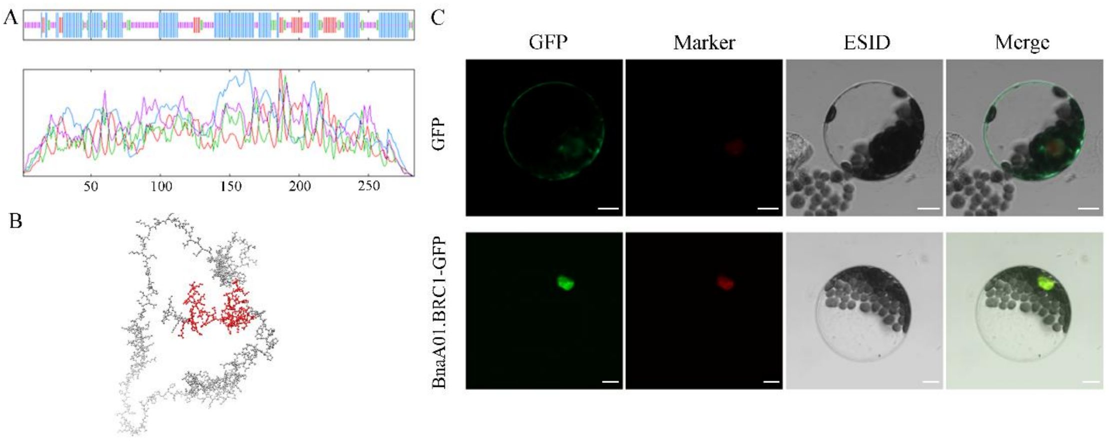

Figure S6. Protein structure and subcellular localization of BnaA01.BRC1. (A) Predicted secondary structure. (B) Predicted tertiary structure with conserved TCP domain. The core TCP functional domain is highlighted in red in the model. (C) Subcellular localization showing nuclear enrichment in *Arabidopsis* protoplasts (Bar = 10 μm).

Table S1. Primers used in this study.

Primers	Primers sequences (F) (5'→3')	Primers sequences (R) (5'→3')	Purpose
BnaA01.BRC1	GTTTCGCAGTTTACAACGATTTT	GCTGCTGCTGCTCTATGGAA	RT-qPCR
BnaA06.RGA1	GCTTTGAACCCGGAGATTAATA	ACTCGACGAATCAACCGCA	
AtBRC1	GGCACAGCAAGATCAAAACGG	AATCCAAGCATGTCTTGTAAGCCAA	
PAN580-*BnBRC1*	GACTAGTATGAAAAATGCCAAGAAACC	CGGGATCCCATATTATGATAGTTGTACAAGAAGTGA	Construction of Subcellular localization vector
M13	GTAAAACGACGGCCAGT	CAGGAAACAGCTATGAC	
PGBKT7-*BnBRC1*	CATGCCATGGATGAAAAATGCCAAGAAACC	CGGGATCCCATATTATGATAGTTGTACAAGAAGTGATG	Construction of yeast two-hybrid vectors
PGADT7-*BnaA06.RGA1*	GGAATTCATGAAGAGGGATCTTCATCAG	CGGGATCCTCAGTGCACAGCCGAGA	
PGADT7-*BnaC09.RGA2*	GGAATTCATGAAGAGAGATCTCCATCAGTTT	CGGGATCCTCAGTGCGCCGCCAA	
p*BnaA01.BRC1*	CCCAAGCTTATGGGTCACTTTTATTGGT	CCCATGGTTTTCGGGTCTTGAGTAGTTG	Construction of the *BnaBRC1* promoter GUS fusion expression vector.
1305.1	CCCCAGGCTTTACACTTTATGCT	GTGTTGATCGGGTACAGACTAGT	
p*BnaA01.BRC1* F-2	CCCAAGCTTTATCTATATTTGATTTCATATTAATTGTAA		
p*BnaA01.BRC1* F-3	CCCAAGCTTAAACAAAACGAATATATACTTCTTTTC		
p*BnaA01.BRC1* F-4	CCCAAGCTTGGTACTCTCGTTCTCTCACTCCA		
p*BnaA01.BRC1* F-5	CCCAAGCTTTGGTACAAGTGTCATTCTCAAA		
p*BnaA01.BRC1* F-6	CCCAAGCTTCCTTATAATGACAATTATTGCTCAC		
p*BnaA01.BRC1* F-7	CCCAAGCTTTCTTCCTTTGAACAACACCATT		
p*BnaA01.BRC1R* -1105	CCCATGGCGTTAATGGTAGAGGAAGGGC		
Desred-*BnBRC1*	CGGGATCCATGAAAAATGCCAAGAAACC	CCCAAGCTTCATATTATGATAGTTGTACAAGAAGTGATG	Construction of the *BnaBRC1* overexpression
Desred	TGGAGAGGACACTAGTGGATCC	CAGGAAACAGCTATGAC	
CRISPR	TATATACAGCTAGAGTCGAAGTAG	TGGGAATCTGAAAGAAGAGAAGCA	Genotyping of *BnaBRC1* knockout lines.
CRISPR-1	TCTTATCTAAGCGATGTGGGACTT	GATGAAGTGGACGGAAGGAAGGAG	
BnaA01g26700D	ATGAAAAATGCCAAGAAACC	CTGCTGAATCCGCCGTGTTT	
BnaA03g34820D	CCATCAGTGAAGACTACATGTTATT	GTTTGAGACTCCTCGCAGC	
BnaC01g34090D	CAAGAAACCAAGCAGAACG	TTGATAGAAAATTGAAACTGTAACT	
BnaC05g51920D	TTTTCTCACTTCGAATCCGT	CTGCCTCTATCCTCGACTGT	
BnaCnng23770D	TCAACACATCAAACAATCATCTT	GGTCGAGTTAGTATTGCTGCC	
