# Supplementary figures and images for: BnaA01.BRC1 Negatively Regulates Branch Number and Responds to Gibberellin Signaling in Brassica napus
Source: Plants (Basel). 2026 Jun 10;15(12):1795. doi: 10.3390/plants15121795 (PMC13306499; doi:10.3390/plants15121795)

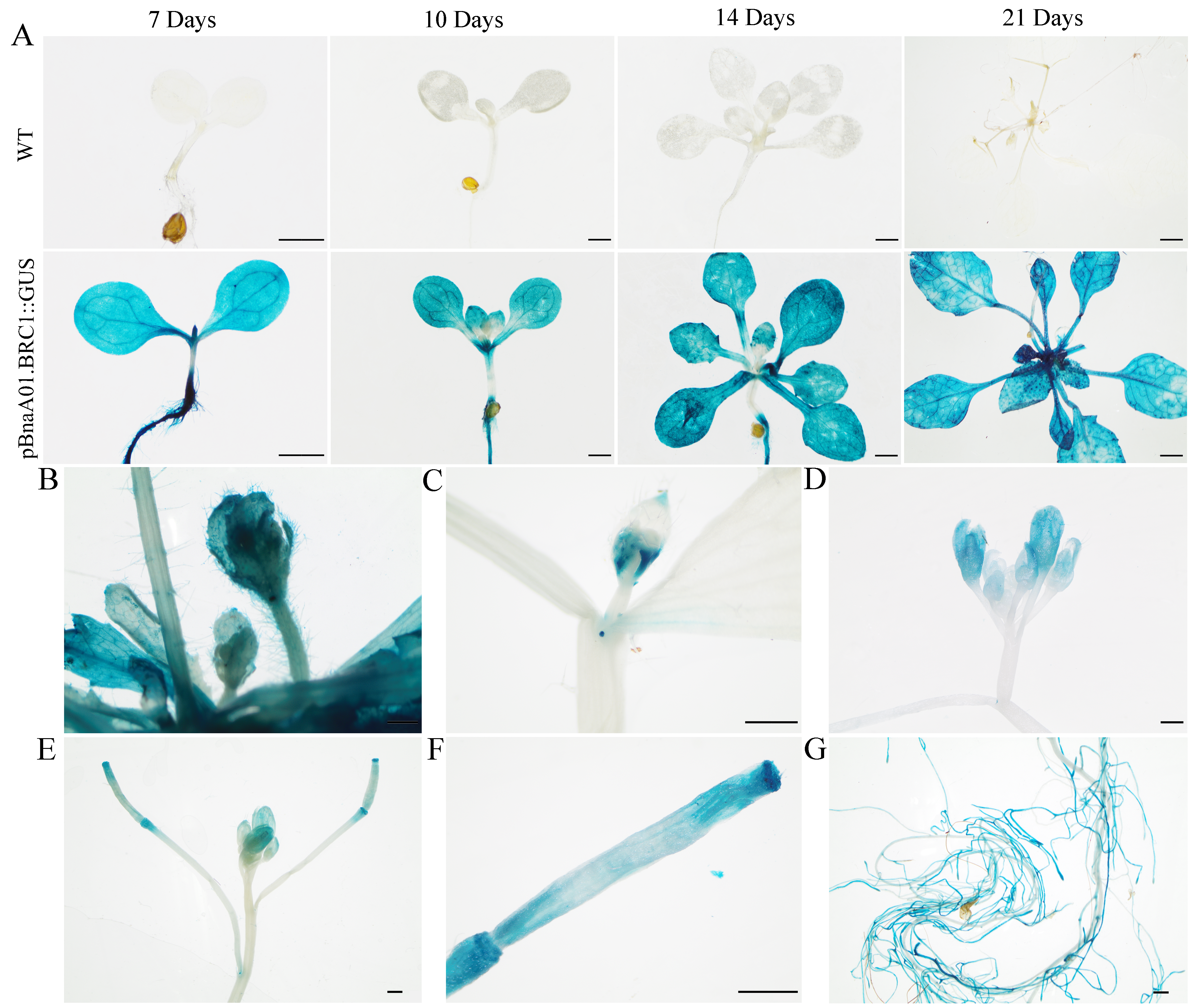

Supplement: Supplementary file 1 [file plants-15-01795-s001.zip › Figure S1.tif]

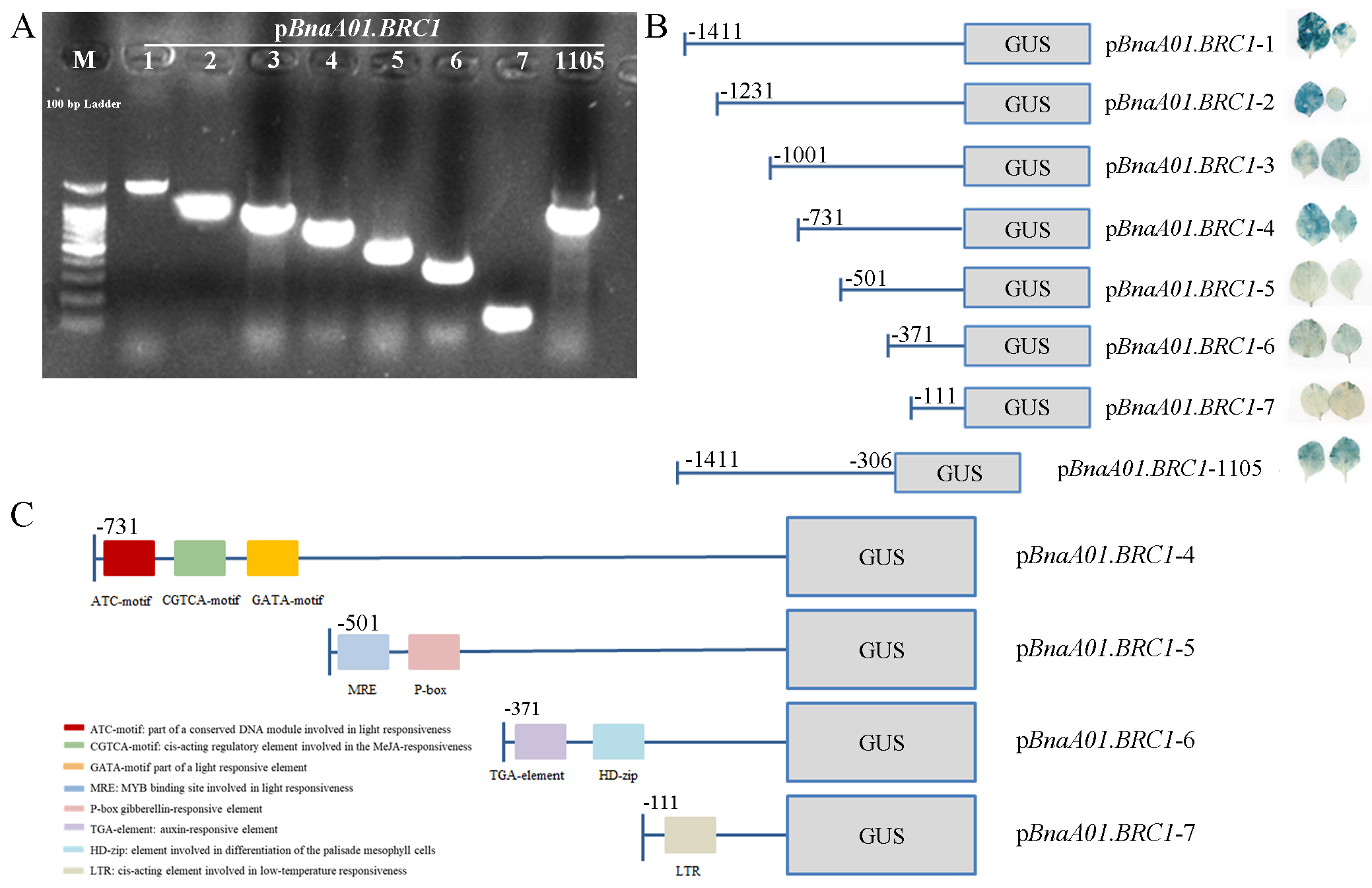

Supplement: Supplementary file 1 [file plants-15-01795-s001.zip › Figure S2.tif]

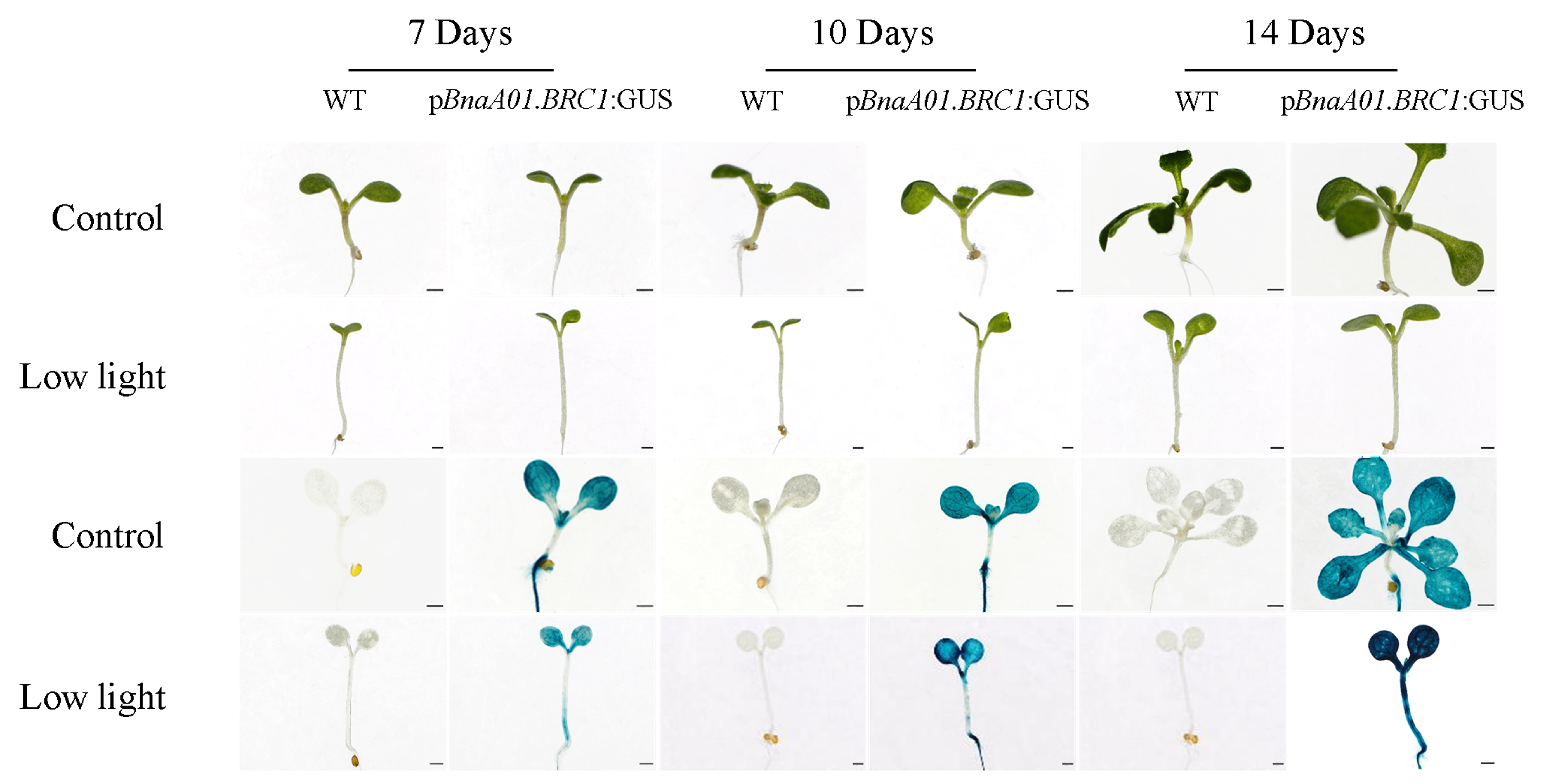

Supplement: Supplementary file 1 [file plants-15-01795-s001.zip › Figure S3.tif]

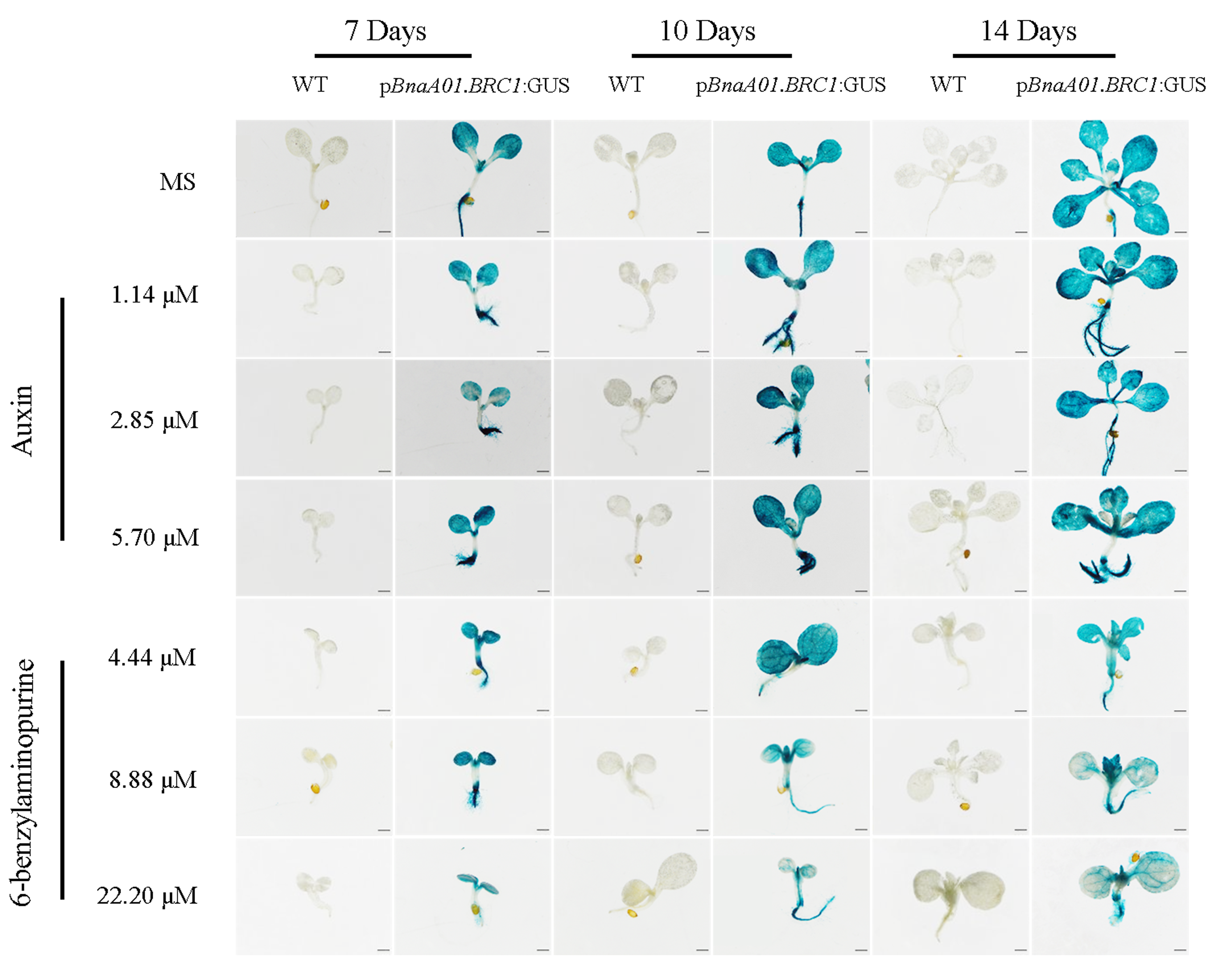

Supplement: Supplementary file 1 [file plants-15-01795-s001.zip › Figure S4.tif]

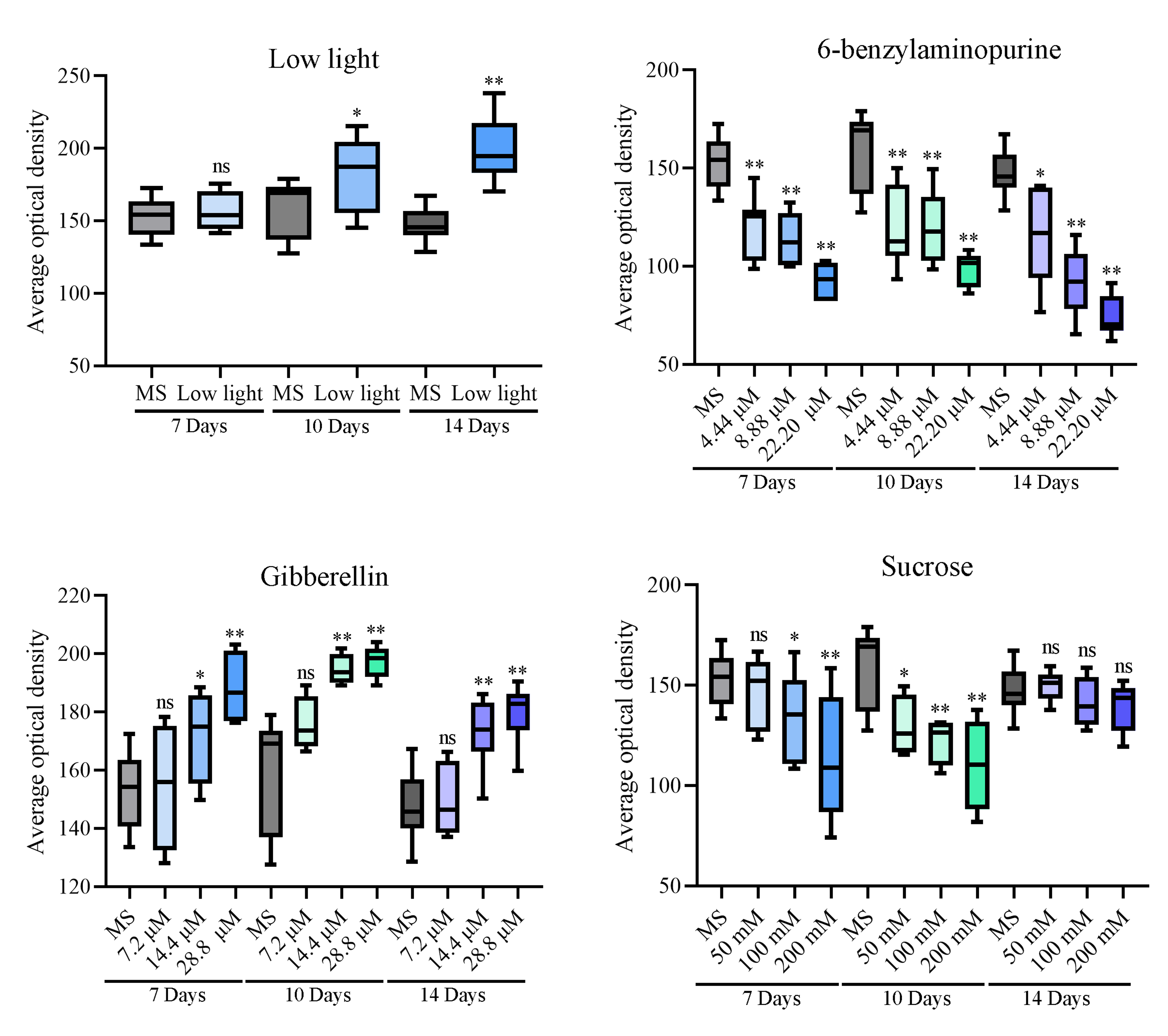

Supplement: Supplementary file 1 [file plants-15-01795-s001.zip › Figure S5.tif]

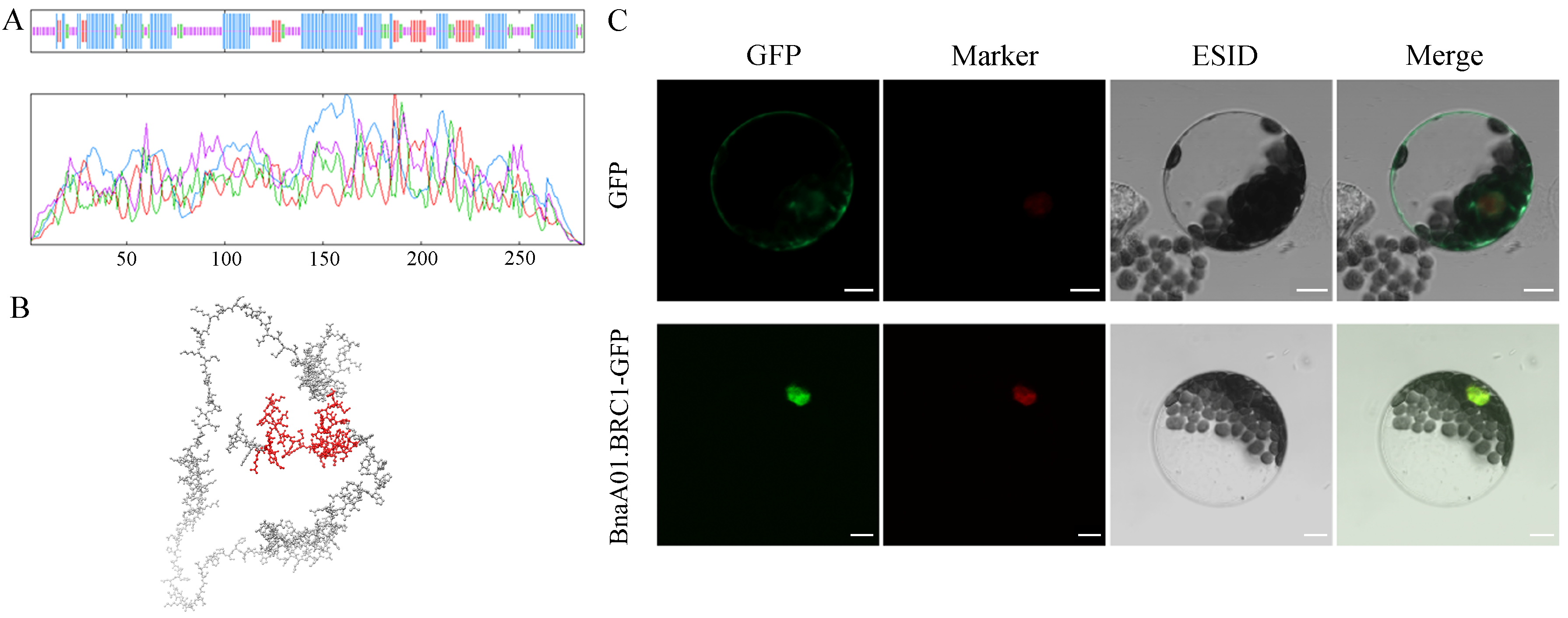

Supplement: Supplementary file 1 [file plants-15-01795-s001.zip › Figure S6.tif]
